# I have the right to carry a backpack: access to the school of children and adolescents in paediatric palliative care in the Veneto Region (Italy)

**DOI:** 10.1186/s13052-025-02167-5

**Published:** 2025-12-02

**Authors:** Anna Santini, Mariangela Rosa, Anna Marinetto, Enrica Grigolon, Luca Giacomelli, Lara Vecchi, Caterina Agosto, Valentina De Tommasi, Franca Benini

**Affiliations:** 1 Pediatric Pain and Palliative Care Service, University Hospital Padua, Padua, Italy; 2 Polistudium srl, Milan, Italy; 3 Department of Women’s and Children’s Health, University of Padua, Padua, Italy

## Abstract

**Background:**

This universal right of access to school applies to all children and adolescents, including paediatric palliative care (PPC) patients. PPC patients display several different levels of disability and cognitive impairment that pose challenges to school inclusion. Therefore, strategies to offer an efficient and tailored school experience that relies on the support of school assistants and support teachers are essential.

**Methods:**

In this retrospective study, data of patients of school age (3–19 years) followed at the Paediatrics Hospice of Padua were collected cross-sectionally from clinical charts, routine visits, and calls to patients and caregivers.

**Results:**

Our results showed that school attendance by students with severe disabilities is remarkably low, especially for those children displaying complex care needs, resulting in a high care burden on families. Notwithstanding, family choices significantly influence school attendance, which may be a consequence of the inadequacy of the services offered by the education system, linked to organisational and structural factors

**Conclusions:**

It is vital to identify the key issues and strengths of the current educational system to develop effective strategies that help families and promote school involvement. Investing in teacher and staff support, along with ongoing training for school personnel, is crucial.

## Background

Access to school is a universal right granted to all children and adolescents [1]. This right applies to children and adolescents with disabilities [2]. However, for them, inclusion into the school system is not easy [2–4], and school experiences of children and adolescents with disabilities are overall worse than those of healthy peers. Children with disabilities tend to have more days of absence from school and overall poorer educational outcomes [5]. Furthermore, the social and relationship life of these children is impaired, with a higher risk of developing depressive symptoms [6–8].

A remarkable proportion of children with a disability require paediatric palliative care (PPC), which is defined as the global care of the child’s body, mind, and spirit, also providing support to the family [9, 10]. PPC should be extended to all children with life-threatening or life-limiting diseases. Remarkably, PPC programs should be instituted when the illness is diagnosed, or even earlier, and should continue regardless of whether a child receives active treatment. A PPC plan should promote cognitive, emotional, and social development [9]. Attending school is a widespread desire of children in PPC and their families [11]. On this line, a PPC plan must consider school access. To this end, the proper allocation of resources is necessary to provide the best possible and tailored school experience for these children, who present with complex and challenging symptoms and have diverse levels of cognitive development [9, 12].

To date, the actual school access of children in PPC has been very poorly investigated. We analyzed the school attendance of children and youth in the Veneto region (Italy), as part of the Veneto Paediatrics Palliative Care Network. We also correlated school attendance with their characteristics, in particular, cognitive development.

## Methods

### Study setting and design

This was a retrospective descriptive study conducted at the Paediatric Hospice of Padua (Padua, Italy; the referral centre for PPC for all the Veneto Region [Northern Italy]), referring to the 2023–2024 academic year. Data were collected from July 2024 to August 2024, corresponding to the school year from September 2023 to June 2024.

The study is non-interventional and based exclusively on de-identified clinical data routinely documented in patient records. Information regarding school attendance and educational support was provided directly by caregivers during routine clinical interactions across various care settings. This information is compiled and updated by professionals of the multidisciplinary care team during scheduled home visits, follow-up phone calls, and planned hospital admissions. These reports were documented by the PPC team in the patients’ medical records and subsequently reviewed for this study. Data protection and confidentiality were ensured in accordance with institutional protocols and the General Data Protection Regulation (GDPR) (EU) 2016/679. The Principal Investigator notified the local Ethics Committee in compliance with national regulations. Informed consent for the use of clinical data for research purposes was obtained from the legal guardians of all patients.

All school-aged patients (3–19 years) followed by the Paediatric Hospice of Padua during the study period were considered eligible. As no formal inclusion or exclusion criteria were applied, the study population reflects the entire cohort of school-aged patients under care. However, patients were excluded from analysis when documentation was insufficient to assess school access. Specifically, missing data were attributed to language barriers (*n* = 8), recent enrollment in PPC during the data collection window without sufficient clinical contact (*n* = 11), or admission to residential care facilities where school access was not documented (*n* = 3).

### Study variables and data analysis

The following variables were considered: the number of hours the patient attends per week, clinical complexity, cognitive and psychosocial functioning, school grades, and the presence and distribution of assistance and study support figures.

The frequency of the disabled child is compared with the weekly hours expected for the rest of the class to which he belongs. Furthermore, it is analysed in detail how the presence of assistance and study support figures is distributed, and this data is compared with the weekly hours expected for the rest of the class to which he belongs. We conducted two separate analyses: one for children not attending school and the other for those attending school regularly. Sub-analyses were conducted with explorative intent to stratify patients according to the phase of schooling.

The patient’s attendance time was calculated based on the full timetable for peers, thus finding the percentage of the timeline attended. This is necessary because each school grade and each institution manages the distribution of the weekly school hours partially independently.

There are three active figures for school support in the Veneto Region (Italy). A school assistant (SA) is a professional specifically trained to manage the clinical care needs of children and young people. On the other hand, the support teacher (ST) is a specialised teacher assigned to the class where a pupil with disabilities is present to promote inclusion and learning strategies. Additionally, a sensory disorders assistant (SDA) helps pupils with visual impairments develop their communication skills.

The functionality of each subject was assessed through the ICF-CY (International Classification of Functioning, Disability and Health-Children and Youth Version) [13, 14]. This conceptual model describes health, functioning, and disability, assessing the ability to perform activities from a predefined list. It relies on a 5-point scale (0 = no impairment and 4 = total disability) [13]. We investigated two items of the ICF-CY: item b-117 (mental functions required to understand and integrate different cognitive abilities and their development) and item b-122 (general mental functions, as they develop over the life span, needed to understand and constructively integrate the mental tasks that lead to the formation of the interpersonal skills required to establish reciprocal social interactions, in terms of both meaning and purpose). For each patient, the ICF-CY assessment was initially performed by at least two experts in PPC who were familiar with the subject, and further discussion was then held within the care team.

Data were analysed using descriptive statistics in MS Excel, including frequency distributions, means, medians, and standard deviations. The analysis was structured to highlight relevant patterns across school grades and ICF severity levels, offering a clear and informative overview of the sample’s educational and clinical characteristics.

## Results

### Study population

As of June 2024, a total of 275 patients were under the care of the Paediatric Hospice of Padua. Of these, 85 were excluded from the analysis as they were not of school age (defined as 3–19 years). An additional 22 patients were excluded due to incomplete documentation regarding school access.

The reasons for missing data included:

- Significant language barriers that hindered effective communication during routine clinical interactions (*n* = 8),
- Recent enrolment in the PPC program during the data collection period, without sufficient time for clinical assessment or contact with families (*n* = 11),
- Admission to residential care facilities where school access was not documented (*n* = 3).

The final study population comprised 168 school-aged patients. Among them, 52 (31%) were not attending school despite being entitled to do so, while 116 (69%) were actively enrolled and attending.

### Patients not attending school

There were 52 patients not attending school, of whom 57.69% were male, with an average age of 10.67. Most subjects have non-oncological disease (50/52; 96.15%), while a small proportion have oncological disease (2/52; 3.85%). Table 1 shows that in most cases (25/52; 48.08%), not attending school was due to the extreme severity of the child’s condition. However, one patient out of four (13/52; 25.00%) did not attend school because of their parents’ perception of the inadequacy of the school system and environment. Some patients (11/52; 21.15%) were attending a daytime facility.

**Table 1:** Descriptive statistics of exemption motivation, clinical complexity, and intellectual and global psychosocial functioning of out-of-school patients

Patients not attending school (tot = 52)			
		Frequency	Percentage
Reason for exemption	Severity-related	25	48.08%
	Parental decision	13	25.00%
	Access facility for disabled people	11	21.15%
	Parental education	2	3.85%
	Lack of assistance	1	1.92%
	**Total**	**52**	**100.00%**
Presence of feeding aids	Yes	44	84.62%
	No	8	15.38%
	**Total**	**52**	**100%**
Presence of ventilation aids	Yes	8	15.38%
	No	44	84.62%
	**Total**	**52**	**100.00%**
Presence of intellectual functioning impairment (ICF-Y b117)	Mild	4	7.69%
	Moderate	2	3.85%
	Severe	7	13.46%
	Complete	39	75.00%
	**Total**	**52**	**100.00%**
Presence of global psychosocial functioning impairment (ICF-Y b122)	Mild	4	7.69%
	Moderate	1	1.92%
	Severe	14	26.92%
	Complete	33	63.46%
	**Total**	**52**	**100.00%**

Regarding clinical complexity, most patients required feeding support (44/52; 84.62%), while 15.38% (8/52) required respiratory support.

Three out of four patients (39/52; 75.00%) showed a complete mental disability, and 63.46% (33/52) had a complete impairment of psychosocial functions.

### Patients attending school

Table 2 depicts the general characteristics of the 116 patients attending schools. Their mean age was 11.08 ± 4.21 years, and the upper age limit was 19. Most patients were affected by non-oncological disease (107/116; 92.24%). Kindergarten is attended by 14 patients (12.07%), Elementary School by 41 (35.34%), Middle School by 27 (23.28%), and High School by 34 (29.31%).

**Table 2:** Socio-demographic descriptive variables of clinical complexity and intellectual and global psychosocial functioning (ICF-Y item b117 e b122) of all school grades

All school grades				
		Mean	Median	SD
**Age**		11.08	11.50	4.21
		**Frequency**	**%**	
**Pathology**	Oncological	4	3.45%	
	Non-oncological	107	92.24%	
	Undiagnosed	5	4.31%	
	**Total**	116	100%	
**Sex**	Male	55	47.41%	
	Female	61	52.59%	
	**Total**	**116**	**100.00%**	
**Type**	Public	114	98.28%	
	Private	2	1.72%	
	**Total**	**116**	**100.00%**	
**School grade**	Kindergarten	14	12.07%	
	Primary school	41	35.34%	
	Middle school	27	23.28%	
	High school	34	29.31%	
	**Total**	**116**	**100.00%**	
**Devices ** (Possible need for more than one device per patient)	Nasogastric tube	5	4.31%	
	Gastrostomy	48	41.38%	
	Tracheostomy	14	12.07%	
	Ventilation: Tracheal tube	12	10.34%	
	Ventilation: non-invasive ventilation	34	29.31%	
	Respiratory physiotherapy	52	44.83%	
	Mucus extractor	77	66.38%	
	AMBU	53	45.69%	
	Oximeter	84	72.41%	
	Postural support	81	69.83%	
**Intellectual functioning: ICF-Y (b117)**	No impairment	39	33.62%	
	Mild	17	14.66%	
	Moderate	11	9.48%	
	Severe	16	13.79%	
	Complete	33	28.45%	
	**Total**	**116**	**100.00%**	
**Global psychosocial functioning: ** **ICF-Y (b122)**	No impairment	45	38.79%	
	Mild	10	8.62%	
	Moderate	19	16.38%	
	Severe	14	12.07%	
	Complete	28	24.14%	
	**Total**	**116**	**100.00%**	

Overall, 10 aids were identified during school time. Most patients required an oximeter (84/116, 72.41%) or postural support (81/116, 69.83%).

Most patients did not show any cognitive or psychosocial impairment (39/116, 33.62% and 45/116, 38.79%, respectively); however, a large proportion of patients showed a complete impairment (33/116, 28.45% and 28/116, 24.14%, respectively).

### Coverage of school attendance and availability of dedicated professionals

Coverage of school attendance is reported in Tables 3 and 4. On average, the disabled student attends 75.60% of the timetable for peers, with 59.42% of the time covered by a school assistant and 69.02% by a support teacher. A provider discussion required specialised in sensory disorders was present for a mean time of 1.66 ± 2.32 h per week. Only 45.69% of patients (53/116) remain in school 100.00% of the time. The school assistant covers half or more of the time in 47.41% of the cases, and the support teacher in 60.34%.

**Table 3:** Statistical indices of the times attended by the sample, care coverage, and presence of study support (all times are indicated on the basis of weekly hours)

	Mean	Median	DS
All school grades			
School hours attended by the patient	23.57	25.00	9.40
School hours by age	31.68	30.00	5.03
% Patient school hours	75.60	91.30	0.30
School assistant (SA) timetable	11.93	12.00	9.50
% Patient school hours covered by SA	59.42	45.08	1.04
Support teacher (ST) timetable	12.38	12.00	7.37
% Patient school hours covered by ST	69.02	55.56	1.47
sensory disorders assistant (SDA)	1.66	2.00	2.32

**Table 4:** Descriptive statistics of the compare of the presence of care figures within the school timetable

		Frequency	Percentage
All school grades			
% Patient school hours	100%	53	45,69%
	< 50%	27	23,28%
	50–99%	89	76,72%
School assistant (SA)	present	96	82,76%
	non present	20	17,24%
	**Totale**	**116**	**100**,**00%**
% Patient school hours covered by SA	*n* < 50%	61	52,59%
	n > = 50%	55	47,41%
	*n* = 100%	16	13,79%
Support teacher (ST)	present	100	86,21%
	non presente	16	13,79%
	**Totale**	**116**	**100.00%**
% Patient school hours covered by ST	*n* < 50%	46	39.66%
	n > = 50%	70	60.34%
	*n* = 100%	20	17.24%
Reader	present	73	62.93%
	non present	43	37.07%
**Total**	**116**		**100.00%**

### School attendance and cognitive/psychosocial function

Tables 5 and 6 summarise information on school attendance for children with various cognitive and psychosocial function levels.

**Table 5:** Descriptive statistics of intellectual functions (ICF-Y-b117) and global psycho-social functioning (ICF-Y-b122) compared with the sample’s school attendance pattern

		ICF_Y b117 (intellectual functioning)		ICF_Y b122 (global psycho-social functioning)	
		Frequency	Percentage	Frequency	Percentage
No impairment/mild impairment					
Attendance mode	Distance education	4	7.14%	3	5.45%
	Home education	1	1.79%	1	1.82%
	Classroom education	51	91.07%	51	92.73%
	Total	56	100.00%	55	100.00%
Moderate					
Attendance mode	Distance education	1	9.09%	1	5.26%
	Home education	1	9.09%	3	15.79%
	Classroom education	9	81.82%	15	78.95%
	Total	11	100.00%	19	100.00%
Severe					
Attendance mode	Distance education	0	0.00%	1	7.14%
	Home education	3	18.75%	2	14.29%
	Classroom education	13	81.25%	11	78.57%
	Total	16	100.00%	14	100.00%
Complete					
Attendance mode	Distance education	1	3.03%	1	3.57%
	Home education	9	27.27%	8	28.57%
	Classroom education	23	69.70%	19	67.86%
	Total	33	100.00%	28	100.00%

**Table 6:** Descriptive statistics of intellectual functions (ICF-Y-b117) and global psycho-social functioning (ICF-Y-b122) compared with times attended by the sample, hours of care coverage and presence of study support

	ICF_Y b117 (intellectual functioning)			ICF_Y b122 (global psycho-social functioning)		
	Mean	Median	SD	Mean	Median	SD
No impairment + mild						
Patient school hours	26.42	28.00	7.95	26.88	28.00	7.23
Full school hours by age	30.30	30.00	4.07	30.31	30.00	4.11
%Patient/full school hours by age	87.80%	100.00%	0.25	89.40%	100.00%	0.23
School assistant (SA) timetable	12.77	12.00	12.00	12.82	12.00	12.00
% patient school hours covered by the SA	69.57%	42.86%	1.46	52.65%	42.86%	0.74
Support teacher (ST) timetable	12.90	14.00	8.04	12.85	13.00	8.10
% patient school hours covered by the ST	76.08%	55.28%	2.10	48.37%	55.00%	0.31
Moderate						
Patient school hours	23.59	20.00	12.00	23.03	20.00	10.62
Full school hours by age	36.00	37.00	4.84	33.21	35.00	5.37
%Patient/full school hours by age	64.90%	62.50%	0.29	68.90%	67.30%	0.27
School assistant (SA) timetable	15.14	15.00	15.00	12.74	10.00	10.00
% patient school hours covered by the SA	65.05%	57.14%	0.33	52.77%	55.56%	0.31
Support teacher (ST) timetable	11.18	10.00	9.2	11.00	10.00	6.47
% patient school hours covered by the ST	48.59%	42.86%	0.38	57.11%	44.44%	0.37
Severe						
Patient school hours	21.72	21.75	9.28	19.61	18.75	11.33
Full school hours by age	30.31	30.00	5.51	31.57	30.50	5.69
%Patient/full school hours by age	72.70%	83.50%	0.28	63.40%	60.60%	0.35
School assistant (SA) timetable	8.47	9.00	9.00	9.50	10.50	10.50
% patient school hours covered by the SA	40.34%	40.00%	0.25	115.08%*	51.67%	2.56
Support teacher (ST) timetable	12.34	12.00	4.24	12.79	14.50	5.82
% patient school hours covered by the ST	66.07%	57.78%	0.33	172.56%*	59.03%	4.12
Complete						
Patient school hours	19.62	21.00	9.58	19.41	21.75	9.31
Full school hours by age	33.23	35.00	5.28	33.38	33.50	5.55
%Patient/full school hours by age	59.90%	61.10%	0.29	59.30%	61.30%	0.29
School assistant (SA) timetable	11.11	10.00	10.00	10.84	10.50	10.50
% patient school hours covered by the SA	49.58%	50.00%	0.33	49.40%	50.00%	0.34
Support teacher (ST) timetable	11.73	10.00	6.90	11.98	12.00	7.32
% patient school hours covered by the ST	65.29%	66.67%	0.33	65.91%	66.67%	0.33

In-person attendance was the most prevalent, regardless of the severity of cognitive impairment; however, a trend toward a reduced frequency of attendance in person as severity increases was observed (from 51/56, 91.07% for patients with no/mild cognitive impairment to 23/33, 69.70% for those with complete impairment). The same trend was observed for the coverage of school schedules (87.80% for subjects with no/mild cognitive impairment vs. 59.90% for those with complete impairment). In comparison, the opposite trend was observed for the support of school assistants (coverage of 69.57% for children with no/mild impairment vs. coverage of 49.58% for those with complete impairment) and support teachers (76.08% vs. 65.29%).

### Subanalysis according to the phase of schooling

Tables 7 and 8 summarize the results of the sub-analyses by phase of schooling. The percentage of total school time attended by patients increases over time: 61.00% at Kindergarten, 69.1% at Primary School, 82.4% at Middle School, and 84.1% at High School.

**Table 7:** Descriptive statistics of the school attended, clinical complexity and psychosocial functioning of the sample divided by school grades

		Kindergarten		Primary School		Middle School		High School	
		Frequency	Percentage	Frequency	Percentage	Frequency	Percentage	Frequency	Percentage
Type	Public	13	92.86%	41	100.00%	26	96.30%	34	100.00%
	Private	1	7.14%	0	0.00%	1	3.70%	0	0.00%
	**Total**	**14**	**100.00%**	**41**	**100.00%**	**27**	**100.00%**	**34**	**100.00%**
Grade	First	1	7.14%	8	19.51%	4	14.81%	14	41.18%
	Second	5	35.71%	10	24.39%	9	33.33%	10	29.41%
	Third	8	57.14%	10	24.39%	14	51.85%	5	14.71%
	Fourth			**7**	17.07%			**1**	2.94%
	Fifth			6	14.63%			4	11.76%
	**Total**	**14**	**100.00%**	**41**	**100.00%**	**27**	**100.00%**	**34**	**100.00%**
Attendance mode	Distance	1	7.14%	1	2.44%	1	3.70%	3	8.82%
	Home	0	0.00%	8	19.51%	5	18.52%	1	2.94%
	Classroom	13	92.86%	32	78.05%	21	77.78%	30	88.24%
	**Total**	**14**	**100.00%**	**41**	**100.00%**	**27**	**100.00%**	**34**	**100.00%**
Devices	Nasogastric tube	0	0.00%	2	4.88%	1	3.70%	2	5.88%
	Gastrostomy	7	50.00%	26	63.41%	7	25.93%	8	23.53%
	Tracheostomy	2	14.29%	6	14.63%	2	7.41%	4	11.76%
	Ventilation: Tracheal tube	2	14.29%	6	14.63%	1	3.70%	3	8.82%
	Ventilation: non-invasive ventilation	2	14.29%	9	21.95%	9	33.33%	14	41.18%
	Respiratory physiotherapy	2	14.29%	22	53.66%	11	40.74%	17	50.00%
	Mucus extractor	10	71.43%	27	65.85%	18	66.67%	22	64.71%
	AMBU	7	50.00%	18	43.90%	12	44.44%	16	47.06%
	Oximeter	8	57.14%	30	73.17%	20	74.07%	26	76.47%
	Postural support	7	50.00%	27	65.85%	21	77.78%	26	76.47%
ICF-Y (b117)	No impairment	2	14.29%	9	21.95%	12	44.44%	16	47.06%
	Mild	0	0.00%	3	7.32%	6	22.22%	8	23.53%
	Moderate	3	21.43%	5	12.20%	0	0.00%	3	8.82%
	Severe	2	14.29%	9	21.95%	2	7.41%	3	8.82%
	Complete	7	50.00%	15	36.59%	7	25.93%	4	11.76%
	**Total**	**14**	**100.00%**	**41**	**100.00%**	**27**	**100.00%**	**34**	**100.00%**
ICF-Y (b122)	No impairment	2	14.29%	10	24.39%	15	55.56%	18	52.94%
	Mild	0	0.00%	2	4.88%	3	11.11%	5	14.71%
	Moderate	5	35.71%	9	21.95%	2	7.41%	3	8.82%
	Severe	2	14.29%	7	17.07%	1	3.70%	4	11.76%
	Complete	5	35.71%	13	31.71%	6	22.22%	4	11.76%
	**Total**	**14**	**100.00%**	**41**	**100.00%**	**27**	**100.00%**	**34**	**100.00%**

**Table 8:** Descriptive statistics comparing school hours and presence of caregivers in the sample divided into school grades

	Kindergarten			Primary School			Middle School			High School		
	Mean	Median	SD	Mean	Median	SD	Mean	Median	SD	Mean	Median	SD
Patient school hours	21.32	21.75	10.60	22.11	24.00	10.39	25.00	27.00	9.10	25.18	27.00	7.67
Full school hours by age	35.36	35.00	4.99	32.51	30.00	6.34	30,39	30.00	3.03	30.18	30.00	3.48
%Patient/full school hours by age	61.00%	61.30%	0.3	69.1	68.80	0.31	82,4	100.00	0.28	84.10	100.00	0.25
School assistant (SA) timetable	13.93	9.50	9.96	11.6	10.00	9.48	11,28	12.00	10.04	12.01	12.00	9.16
% patient school hours covered by the SA	66.75	66.00%	0.30	58.48	42,86	0.82	38.95	44.44	0.31	73.79	43.65	1.67
Support teacher (ST) timetable	8.32	10.00	7.00	13.38	12.00	7.63	13.20	12.50	7.57	12.01	12.00	6.72
% patient school hours covered by the ST	41.82%	34.77%	0.44	64.92	60.00	0.33	56.49	55.56	0.31	95.12	54.25	2.67
Reader (hours)	1.50	2.00	1.02	1.9	2	3.12	1.41	2.00	1.34	1.62	2.00	2.23

Regarding the Kindergarten, public school is a clearly privileged choice (92.86%), and the method of in-person attendance (92.86%). Public school is a clearly privileged choice regarding the level of nursery school (92.86%) and the technique of in-person attendance (92.86%). The devices required at school vary, with aspirators (71.43%) and pulse oximeters (57.14%) being the most prevalent. In half of the cases, the patient uses a postural aid, such as the Auxiliary Manual Breathing Unit (AMBU), and a gastrotomy aid at school. The impairment of intellectual functions is complete in 50% of patients, while the impairment of global psycho-social functions is moderate in 35.71% and complete in 35.71% of patients.

Primary school is attended in the public form by all patients; the second and third classes are the largest (both 24.39%). The frequency mode is present but not completely (78.05%). The aids are varied; it should be noted that 53.66% of children bring aids for respiratory gymnastics to school, and 73.17% bring a pulse oximeter. More than half, 65.85%, receive postural assistance at school. The impairment of intellectual functions is complete for 36.59%, and of global psychosocial functions, it is complete for 31.71%.

Most Middle School students chose the public option (96.30%), with the highest turnout in the third year (51.85%). The presence method is the most chosen (77.78%). The aspirator (66.67%) and the pulse oximeter (74.07%) are the most common devices, and the wheelchair is a standard postural device for 77.78% of children. The impairment of intellectual functions is not present in 44.44% of cases, while that of global psychosocial functions is not present in 44.44% of cases.

Secondary school is attended entirely in its public form, with most students enrolled in the first year (41.18%). The most common attendance mode is presence (88.22%). The oximeter (76.47%) is the most common device alongside postural devices. The impairment of cognitive functions is not present in 47.06% of cases, and the same is true for global psychosocial functions, which are absent in 52.94% of cases. The devices required at school vary, with predominance of aspirators (71.43%) and pulse oximeters (57.14%). In half of the cases, the patient uses a postural aid, including the AMBU and gastrotomy aids, at school. The most frequent impairment of intellectual functions is complete (50%), while the impairment of global psycho-social functions is spread between medium (35.71%) and complete (35.71%).

The nursery school pupil attends 61.00% of the full timetable by age, with 66.75% of it covered (13.93 h per week) by the school assistant and 41.82% by the support teacher (8.32 h per week). The sensory disorders assistant is present for an average of 1.5 h per week.

By age, the primary school pupil attends 69.10% of the full timetable, with 58.48% (11.60 h per week) covered by the school assistant and 69.42% (13.38 h per week) by the support teacher.

The Middle School pupil attends 82.4% of the full timetable, with the school assistant present for 38.95% (11.28 h per week) and the support teacher for 56.49% (13.20 h per week). Co-presence does not occur, while the sensory disorders assistant is present for an average of 1.41 h per week.

The High School patient attends 84.1% of the time scheduled for those of the same age, the school assistant is present for 73.79% of the time (12.01 h per week), and the support teacher is present for 95.12% (12.01 h per week).

## Discussion

Although the right to education for all is enshrined in international and national regulations, it is not always fully implemented in the case of students with severe disabilities, and school inclusion represents a complex and constantly evolving area of research [15]. This study aimed to analyse the specific characteristics of this population, and our preliminary results indicate that a significant proportion of students with severe disabilities do not attend school, resulting in a high care burden on families [16]. The situation worsens as the complexity of the care needs of these children increases, and the non-attendance at school is also frequently a family choice. Caregivers often express concerns about the safety of their child within the school environment, fearing that the student may be passively supervised without meaningful engagement or may not receive a standard of care comparable to that provided at home. Such perceptions indicate a limited confidence in the school system’s capacity to deliver adequate assistance and to establish effective, individualized interactions with children and adolescents presenting with complex disabilities. Consequently, school non-attendance in these circumstances is more appropriately interpreted as a response to perceived systemic inadequacies, rather than as a purely voluntary or culturally motivated decision.

Based on these results, the hypothesis that the service offered by the education system is inadequate, linked to organisational factors and the presence of architectural barriers, cannot be excluded [17, 18]. Besides this, feeding and ventilation devices often pose limitations that do not allow for total attendance, regardless of the impairment of global intellectual or psycho-social functions. Therefore, they represent significant obstacles to inclusion. The analysis also showed that impairment of intellectual and psycho-social functions leads to a lesser presence at school but highly activates both the assistance figures and the support teachers. However, the assistance figures do not cover the entire time of school attendance, leaving the total management of the child in their absence to the support teacher.

In the context of school inclusion, special attention should be paid to students with severe disabilities who attend secondary schools due to the challenges they face while transitioning into adult life. In this context, the scarcity of projects aimed at promoting autonomy and independence, combined with the high demand for caregivers’ presence, limits the possibilities of social and work inclusion [19]. Furthermore, the fact that school attendance is often lower than that of peers [20], as confirmed in this study, has a negative impact on the development of social and cognitive skills.

Investing in the continuous training of school staff is crucial for promoting the inclusion of students with severe disabilities and providing them with an effective educational plan. Additionally, it is essential to maintain a consistent and coordinated effort from all stakeholders and ensure a fair allocation of resources to mitigate inequalities among educational institutions. Thanks to their specialized skills, healthcare providers can play a crucial role in supporting these children and their families, helping to create a more inclusive and welcoming school environment for all.

## Conclusions

The variability of inclusion experiences highlights the need for a more systematic and personalised approach to respond to each student’s specific needs. Further research is therefore necessary to delve deeper into the determinants of school inclusion and to identify effective and sustainable intervention models that can support families and encourage school participation.

## Data Availability

Data are available on request to the corresponding author.
